# Trends and Themes in *Mycoplasma genitalium* Consultations Received Through the Sexually Transmitted Diseases Clinical Consultation Network 2015–2024

**DOI:** 10.1093/ofid/ofag041

**Published:** 2026-03-30

**Authors:** Jacob McLean, Jacqueline Sherbuk, Wyatt Hanft, Kelly A Johnson, Barbara Wilgus, Destiny Kelley, Nicholas Van Wagoner, William M Geisler

**Affiliations:** Division of Infectious Diseases, Columbia University Vagelos College of Physicians and Surgeons, New York, NY, USA; Division of Infectious Disease and International Health, University of South Florida Morsani College of Medicine, Tampa, FL, USA; Department of Family and Community Medicine, University of California San Francisco, San Francisco, CA, USA; California Prevention Training Center, San Francisco, CA, USA; California Prevention Training Center, San Francisco, CA, USA; Division of Infectious Diseases, University of California SanFrancisco, San Francisco, CA, USA; Department of Medicine, Johns Hopkins University School of Medicine, Baltimore, MD, USA; Denver Prevention Training Center, Public Health Institute at Denver Health, Denver, CO, USA; Department of Medicine, University of Alabama at Birmingham Heersink School of Medicine, Birmingham, AL, USA; Department of Medicine, University of Alabama at Birmingham Heersink School of Medicine, Birmingham, AL, USA

**Keywords:** *Mycoplasma genitalium*, sexually transmitted infections, pregnancy, referral and consultation

## Abstract

**Background:**

*Mycoplasma genitalium* is an emerging sexually transmitted pathogen. *M. genitalium* is often asymptomatic but can cause urethritis, is associated with cervicitis and pelvic inflammatory disease, and possibly infertility and premature labor. Although multiplex sexually transmitted infection (STI) testing platforms that include *M. genitalium* are available, screening for asymptomatic infection is not recommended as its potential clinical impact is unknown. Leveraging the STD Clinical Consultation Network (STDCCN)—an STI consultation service for healthcare and public health professionals in the USA—our study aimed to identify training needs by analyzing temporal and thematic trends in *M. genitalium* consultations.

**Methods:**

We reviewed STDCCN consults referencing “*Mycoplasma*,” “*genitalium*,” “m gen” and “mgen” between 2015 and 2024. Consult data were extracted, reviewed by 2 groups of 2 authors (with another reviewer adjudicating disagreements), and categorized by theme(s) and patient characteristics.

**Results:**

336 *M. genitalium* consults were identified, of which 75% occurred after 1 January 2023. Common themes were management in pregnancy (42%) and treatment failure (35%). Pregnant patients were less likely than nonpregnant patients to have symptoms reported. Consults pertaining to *M. genitalium* versus other topics were more likely to come from providers (physicians, nurse practitioners, nurse midwives, and physician assistants) than individuals in other roles, and more likely to come from individuals in private practices or women's health clinics than other practice settings.

**Conclusions:**

STDCCN observed a substantial increase in *M. genitalium* consultations starting in 2023. This rise was likely driven by *M. genitalium* treatment failures and detection of *M. genitalium* in asymptomatic pregnant people. This study highlights the need for provider education about appropriate use of *M. genitalium* diagnostics, indications for *M. genitalium* screening/testing, and management of *M. genitalium* treatment failures.

*Mycoplasma genitalium* is an emerging sexually transmitted bacterial pathogen, found in the United States to be more prevalent than gonorrhea in the general population [1] and of similar or higher prevalence than chlamydia in clinic-based populations [2]. Clinically, *M. genitalium* causes urethritis in men and is associated with cervicitis, PID, and possibly infertility as well as adverse pregnancy/perinatal outcomes in women [3–8]. *M. genitalium* also causes acute urethral syndrome in women and is associated with human immunodeficiency virus 1 (HIV-1) acquisition regardless of sex [3–8]. *M. genitalium* infection is often asymptomatic [1, 9], but the natural history of asymptomatic infection is poorly understood; it remains to be elucidated whether persistent (untreated) asymptomatic infection has clinical consequences [10].

*M. genitalium* testing practices have evolved in recent years based in part on availability of *M. genitalium* diagnostic tests and recommendations from the Centers for Disease Control and Prevention (CDC). The 2015 CDC Sexually Transmitted Diseases Treatment Guidelines included mention of *M. genitalium* in the Emerging Issues section, highlighting the clinical syndromes for which this pathogen should be suspected/considered and stating that a nucleic acid amplification test (NAAT) was the preferred *M. genitalium* detection method. At the time, however, there were no Food and Drug Association (FDA)-cleared *M. genitalium* NAATs available, so provider access to testing remained limited [11]. The FDA subsequently granted clearance for the first *M. genitalium* NAAT in 2019 [12], with additional NAATs (both FDA cleared and noncleared) becoming commercially available thereafter. Available NAATs typically combine testing for *M. genitalium* with 1 or more other sexually transmitted infections (STIs) that can be detected in the urogenital tract. In the most recent CDC 2021 STI Treatment Guidelines, there is a dedicated *M. genitalium* section that recommends *M. genitalium* testing for recurrent urethritis or cervicitis and suggests considering *M. genitalium* testing in pelvic inflammatory disease [13]. The CDC still does not recommend screening for asymptomatic *M. genitalium* infection.

For over 40 years, CDC-supported sexually transmitted diseases (STD)/HIV Prevention Training Centers (collectively known as the National Network of STD Clinical Prevention Training Centers [NNPTC]) have provided comprehensive, evidence-based STD education and training to clinical providers and public health professionals, with the goal of advancing STD prevention and control efforts [14]. The STD Clinical Consultation Network (STDCCN) is a major NNPTC initiative that was launched nationally in 2015 and is offered by all 8 regional STD clinical PTCs within the NNPTC as a means for free STI consultation with STI experts [15]. Individuals submitting requests to the STDCCN complete a brief online form collecting information about the requester, and then submit a free-text consult question. In recent years, consultants responding to STDCCN requests anecdotally observed an increase in requests related to *M. genitalium*—particularly consults related to *M. genitalium* testing/screening practices that are outside of CDC guideline recommendations. This prompted the development of a study, presented here, that used STDCCN data to investigate general, geographic, and temporal trends in *M. genitalium* consultations across PTCs over time to help identify gaps in provider *M. genitalium* knowledge, and to guide future *M. genitalium* education and training needs for clinicians and public health professionals.

## METHODS

We performed a retrospective review of STDCCN consult requests submitted from 15 June 2015 to 30 June 2024 using data obtained from the NNPTC. Archived STDCCN consult data were extracted for the dates noted and provided in database form from the NNPTC Coordination Center in Denver, CO. Location, occupation, and practice setting of the submitting individual, verbatim text of the submitted consult question, consult submission date, and consult topic assigned by the responding PTC (including specific pathogen as appropriate) were included for analysis. To ensure capture of all requests pertaining to *M. genitalium*, consult question text was also systematically searched for “*Mycoplasma*,” “*genitalium*,” “mgen,” and “m gen.” All consults identified by this search, as well as those labeled by consultants as pertaining to *M. genitalium*, were included for manual review.

Two groups of 2 study authors (J. S. and W. H. or B. W. and K. A. J.) independently reviewed the text of each potential *M. genitalium* consult question, and abstracted pertinent data into a REDCap database. An additional study author (N. V. W.) adjudicated any discrepancies between data reviewers to create the final data set. For each consult, reviewers identified consult type and the central consult question/theme(s); consult types and questions/themes are listed in Table 1. Multiple themes could be assigned to a single consult. Consults regarding *M. genitalium* treatment failure were subcategorized based on presence/absence of symptoms and results of test of cure (TOC) if provided. For consults involving a clinical case, de-identified patient information and clinical details were abstracted if included in the consult question, including sex, pregnancy status and trimester, presence and type of symptoms, and any specifically mentioned clinical syndrome. In determining sex, we labeled patients as male when male anatomy was referenced (eg, penile discharge) or when masculine pronouns (“his/him”) were used, unless a non-*cis* gender identity was specified. We used the same rationale for labeling female sex. Consult questions that were found to pertain to *Mycoplasma hominis*, unspecified *Mycoplasma* species, or non-*Mycoplasma* pathogens were excluded from the analysis of *M. genitalium* consults.

**Table 1.** Demographic and Clinical Characteristics of Patients Described in *Mycoplasma Genitalium* Clinical Consults

Category	(N = 296)
Sex	
Female	174 (58.8)
Male	107 (36.1)
Not specified	15 (5.1)
Symptomatic at time of consult submission	
Yes	156 (52.7)
No	43 (14.5)
Not specified	97 (32.8)
Symptomatic at any time during clinical course	
Yes	174 (58.8)
No	28 (9.5)
Not specified	94 (31.8)
STI co-infection present	
Yes	41 (13.9)
No	255 (86.1)
Pregnancy status	
Not pregnant	165 (55.7)
Pregnant	131 (44.3)
1st trimester	38
2nd trimester	42
3rd trimester	24
Not specified	27

We performed a descriptive analysis of all STDCCN consults assessing volume over time, proportion of total consults pertaining to *M. genitalium*, as well as occupation, workplace setting, and location of the submitting individual. We compared consults for *M. genitalium* versus other topics by occupation and workplace setting of the submitting individual using Fisher's Exact Test. Within the subset of *M. genitalium* consults, we performed descriptive analyses of consult types and themes, patient characteristics, and clinical features. We assessed association between sex and symptom status as well as pregnancy and symptom status using χ^2^ tests. Statistical analysis was done using R software, version 4.4.3.

## RESULTS

8204 consult requests were submitted to the STDCCN over the study period, of which 407 (5.0%) were identified on initial screening as possibly pertaining to *M. genitalium*. After review, 71 entries were excluded from the analysis of *M. genitalium* consults: 4 duplicates, 4 nonconsult (eg, “test”) entries, 4 consults for non-*Mycoplasma* pathogens, 4 consults for M. hominis, and 55 consults for *Mycoplasma* without a specified species. 336 requests specifically pertaining to *M. genitalium* were thus included for analysis, representing 4.1% of total requests during the study period.

**Figure 1.**
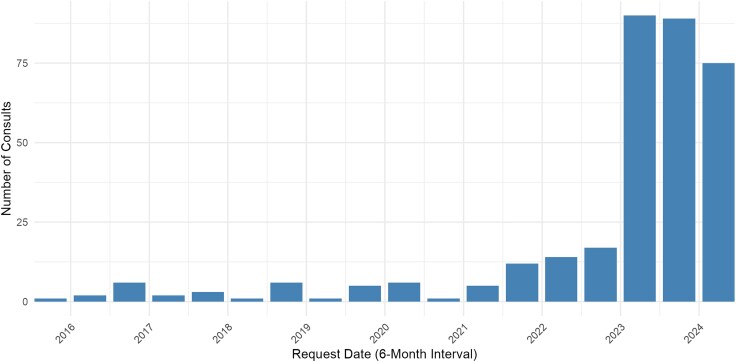
Semiannual totals of STDCCN consults for *Mycoplasma genitalium,* 15 June 2015 to 30 June 2024.

**Figure 2.**
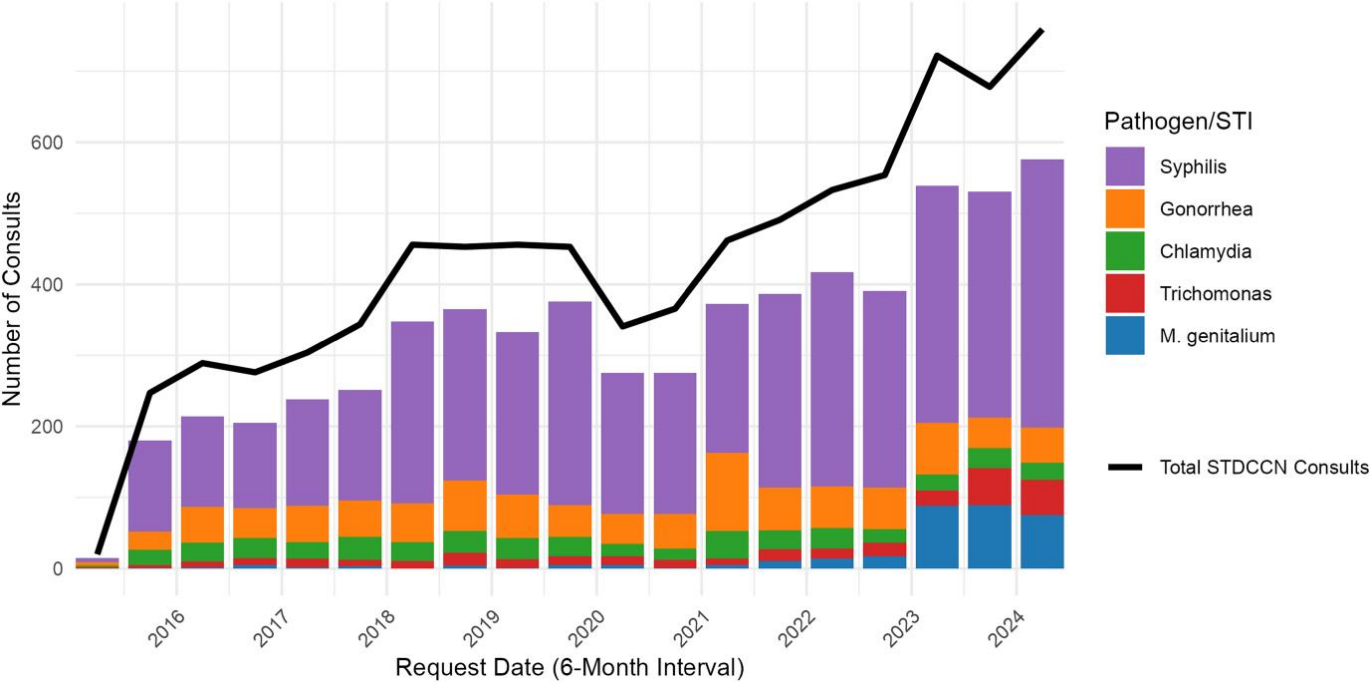
Semiannual totals of STDCCN consults for the 5 most common STI pathogens, 15 June 2015 to 30 June 2024.

The majority of *M. genitalium* consults (254/336, 75.6%) were submitted on or after 1 January 2023 (Figure 1). *M. genitalium* consults comprised a significantly greater proportion of overall STDCCN consults submitted after 1 January 2023 compared with before that date (254/2159, 11.8% vs 82/6045, 1.4%; *P* < .01). Among consults submitted after 1 January 2023, *M. genitalium* consults were second in frequency only to syphilis consults (1031/2159, 47.8%) (Figure 2). Among *M. genitalium* consults, 88.1% (296/336) were for clinical cases, followed by general questions (9.5%) and requests for technical assistance (2.1%). One consult submission had no identifiable question.

Of the 296 patients described in the *M. genitalium* clinical case consults, 58.8% were identified as female, 36.1% as male, and 5.1% had no identifiable sex. No patients were described as transgender or gender nonbinary. Patients were identified as pregnant in 44.3% of cases. With regard to symptom status, 58.8% of clinical consults described symptoms either at the time of submission or at a previous point in the clinical course. There was no mention of current or prior symptoms in 31.8% patients, and 9.5% were explicitly noted to have never had symptoms. A slightly higher proportion (14.5%) of cases were noted to be asymptomatic at the time of submission (Table 1). In 174 clinical cases with specified symptoms, the most common among males and females were urethral discharge (52.2%), and vaginal discharge (48.1%), respectively. In the 54 clinical consults naming specific clinical syndromes, urethritis was the most common among males (94.7%) and vaginitis among females (60.0%) (Table 2).

**Table 2.** Specific Symptoms and Clinical Syndromes Reported in *Mycoplasma Genitalium* Clinical Consults

Symptom	Male (N = 90)	Female (N = 81)	Sex Not Specified (N = 3)
Urethral discomfort	46 (51.1)	8 (9.9)	1 (33.3)
Urethral discharge	47 (52.2)	0 (0.0)	0 (0.0)
Testicular pain	8 (8.9)	0 (0.0)	0 (0.0)
Vaginal discomfort	0 (0.0)	11 (13.6)	0 (0.0)
Vaginal discharge	0 (0.0)	39 (48.1)	0 (0.0)
Vaginal odor	0 (0.0)	7 (8.6)	0 (0.0)
Postcoital or intramenstrual bleeding	0 (0.0)	1 (1.2)	0 (0.0)
Pelvic pain	0 (0.0)	8 (9.9)	0 (0.0)
Rectal discomfort	2 (2.2)	0 (0.0)	0 (0.0)
Anal discharge	1 (1.1)	0 (0.0)	0 (0.0)
Other symptom	9 (10.0)	24 (29.6)	0 (0.0)
Symptom not specified	18 (20.0)	16 (19.8)	2 (66.6)

Syndrome	Male (n = 38)	Female (n = 15)	Sex Not Specified (n = 1)
Urethritis	36 (94.7)	0 (0.0)	0 (0.0)
Vaginitis	0 (0.0)	9 (60.0)	0 (0.0)
Pelvic inflammatory disease	0 (0.0)	4 (26.7)	0 (0.0)
Epididymitis	2 (5.2)	0 (0.0)	0 (0.0)
Proctitis	2 (5.2)	0 (0.0)	0 (0.0)
Other	2 (5.2)	0 (0.0)	1 (100.0)
Cervicitis	0 (0.0)	2 (13.3)	0 (0.0)

Within the 296 clinical consults, female patients were more likely than male patients to be asymptomatic or have no symptoms reported (93/174, 53.4% vs 17/107, 15.9%, *P* < .001). Among female patients, those who were pregnant were more likely than those who were not to be asymptomatic or have no symptoms reported (76/131, 58.0% vs 17/43, 39.5%, *P* = .035).

The most common *M. genitalium* consult themes were management of *M. genitalium* in a pregnant person (42%), management of an initial/new episode of *M. genitalium* (36.0%), and management of *M. genitalium* treatment failure (35.4%) (Table 3). Among the 119 consults related to *M. genitalium* treatment failure, 47.1% noted both persistent symptoms and a positive TOC, 18.5% noted persistent symptoms without mention of TOC, 16.8% noted a positive TOC without mention of symptoms, and 11.8% noted a positive TOC in the absence of symptoms (Table 4).

**Table 3.** Themes of All *Mycoplasma Genitalium* Consults Submitted to the STDCCN

Theme (N = 336)	
Management of Mgen in a pregnant patient	141 (42.0)
Management of new/initial Mgen episode (not treatment failure)	121 (36.0)
Management of Mgen treatment failure	119 (35.4)
Management of Mgen with antibiotic side effects or allergies	43 (12.8)
Management of partner/contact of an infected person	41 (12.2)
STI co-infection present^a^	41 (12.2)
Other consult theme	40 (11.9)
Decision to perform post-treatment testing/test of cure	20 (6.0)
Access to Mgen resistance testing	19 (5.7)
Management of suspected Mgen in the absence of confirmatory testing	13 (3.9)
Interpreting initial Mgen positive result in an asymptomatic person	10 (3.0)
Access to medications for Mgen treatment	9 (2.7)
Decision to screen an asymptomatic person for Mgen	7 (2.1)
Access to Mgen testing (excluding resistance testing)	6 (1.8)
Nature of question not clear from consult text	5 (1.5)
Interpreting initial Mgen positive result in a symptomatic person	3 (0.9)

Abbreviations: Mgen, *Mycoplasma genitalium*; STI, sexually transmitted infection.

^a^Included STIs: syphilis, *N. gonorrhea*, *C. trachomatis, T. vaginalis*, bacterial vaginosis, vulvovaginal candidiasis, and genital herpes.

**Table 4.** Symptom Status and Test of Cure Results in *Mycoplasma Genitalium* Clinical Consults Describing Treatment Failure

Symptoms/TOC Results (n = 118)	
Persistent symptoms/positive TOC	56 (47.5)
Persistent symptoms/no TOC	22 (18.6)
Unspecified symptom status/positive TOC	20 (16.9)
Asymptomatic/positive TOC	14 (11.9)
Persistent symptoms/negative TOC	3 (2.5)
Unspecified treatment failure	3 (2.5)

Abbreviation: TOC, test of cure.

Among clinical consults in which patient sex was known, a higher proportion of consults for male patients compared with female patients involved Mgen treatment failure (75% vs 19%, *P* < .001), Mgen management in the context of medication allergies/side effects (20% vs 9.2%, *P* = .012), and decision to perform a TOC (9.3% vs 3.4%, *P* = .038). Among female patients, these same themes were more common among nonpregnant patients compared with pregnant ones. A higher proportion of consults for female patients compared with males related to new/initial Mgen infection (56% vs 11%, *P* < .001) as well as STI co-infection (22% vs 1.9%, *P* < .001), with no difference in frequency between pregnant and non-pregnant female patients. Themes of Mgen treatment without confirmatory testing and access to Mgen resistance testing were more common among male than female patients, with no difference between pregnant and nonpregnant female patients (Table 5).

**Table 5.** Themes of *Mycoplasma Genitalium* Clinical Consults Submitted to the STDCCN Compared by Sex and by Pregnancy Status

Theme	Patients With Known Sex (N = 281)			Female Patients (N = 174)		
	Female (N = 174)	Male (N = 107)	*P* Value	Not Pregnant (N = 43)	Pregnant (N = 131)	*P* Value
Management of new/initial Mgen episode (not treatment failure)	97 (56%)	12 (11%)	<.001	20 (47%)	77 (59%)	.2
Management of Mgen treatment failure	33 (19%)	80 (75%)	<.001	15 (35%)	18 (14%)	.002
Management of Mgen with antibiotic side effects or allergies	16 (9.2%)	21 (20%)	.012	14 (33%)	2 (1.5%)	<.001
Management of partner/contact of an infected person	21 (12%)	12 (11%)	.8	10 (23%)	11 (8.4%)	.009
STI co-infection present^a^	39 (22%)	2 (1.9%)	<.001	11 (26%)	28 (21%)	.6
Decision to perform post-treatment testing/test of cure	6 (3.4%)	10 (9.3%)	.038	4 (9.3%)	2 (1.5%)	.033
Access to Mgen resistance testing	0 (0%)	14 (13%)	<.001	0 (0%)	0 (0%)	>.9
Management of suspected Mgen in the absence of confirmatory testing	1 (0.6%)	7 (6.5%)	.006	1 (2.3%)	0 (0%)	.2
Interpreting initial Mgen positive result in an asymptomatic person	8 (4.6%)	1 (0.9%)	.2	4 (9.3%)	4 (3.1%)	.10
Access to medications for Mgen treatment	2 (1.1%)	4 (3.7%)	.2	2 (4.7%)	0 (0%)	.060
Decision to screen an asymptomatic person for Mgen	0 (0%)	4 (3.7%)	.020	(0%)	0 (0%)	>.9
Access to Mgen testing (excluding resistance testing)	0 (0%)	2 (1.9%)	.14	0 (0%)	0 (0%)	>.9
Nature of question not clear from consult text	1 (0.6%)	0 (0%)	>.9	0 (0%)	1 (0.8%)	>.9
Interpreting initial Mgen positive result in a symptomatic person	3 (1.7%)	0 (0%)	.3	1 (2.3%)	2 (1.5%)	>.9

Abbreviations: STI, sexually transmitted infection; Mgen, *Mycoplasma genitalium*.

^a^Included STIs: syphilis, *N. gonorrhea*, *C. trachomatis, T. vaginalis*, bacterial vaginosis, vulvovaginal candidiasis, and genital herpes.

**Figure 3.**
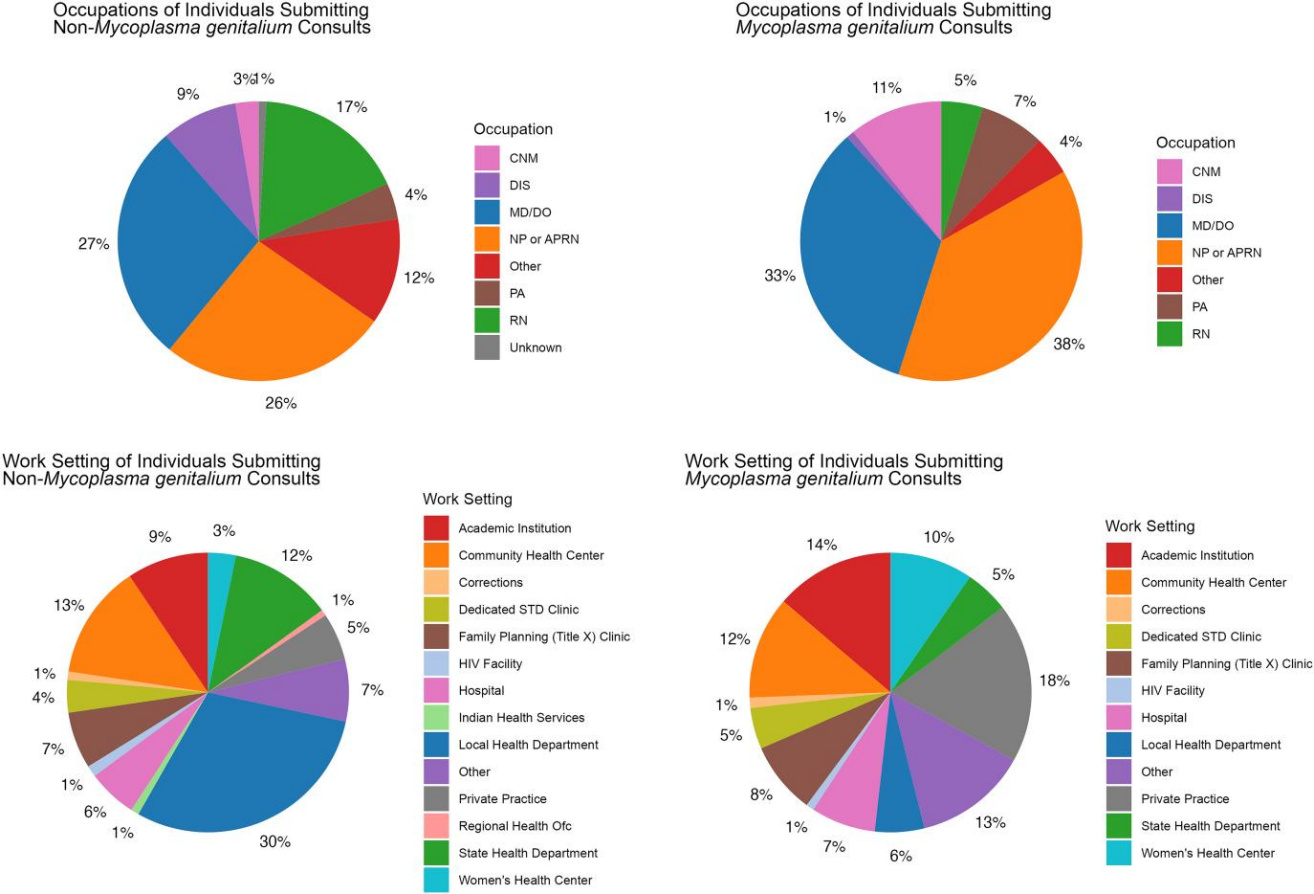
Occupation and work setting of individuals submitting *Mycoplasma genitalium* and non-*Mycoplasma genitalium* consults. Abbreviations: APRN, advanced practice registered nurse; CNM, certified nurse midwife; DIS, disease intervention specialist; HIV, human immunodeficiency virus; MD/DO, medical doctor/doctor of osteopathy; Mgen, *Mycoplasma genitalium*; NP, nurse practitioner; PA, physician assistant; RN, registered nurse; STD, sexually transmitted disease.

The most common occupations of individuals requesting consults for *M. genitalium* were nurse practitioner (NP) or advanced practice registered nurse (APRN) (N = 128, 38%), medical doctor/doctor of osteopathy (MD/DO) (N = 112, 33%), and certified nurse midwife (CNM) (N = 36, 11%). The distribution of requester occupations differed significantly between consults regarding *M. genitalium* and consults for other pathogens/topics (*P* < .01). Consults pertaining to *M. genitalium* were more likely to have come from a provider (MD, DO, NP, APRN, CNM, or physician assistant) versus nonprovider (registered nurse, licensed practical nurse, medical assistant, disease intervention specialist, or other occupation) than consults for topics other than *M. genitalium* (301/336, 90% vs 4740/7868, 60%, *P* < .01). The most common work settings for individuals submitting *M. genitalium* consults were private practice (62/336, 18%), academic institution (46/336, 14%), and work settings entered as “Other” (44/336, 13%). The overall distribution of work settings also differed significantly between consults regarding *M. genitalium* and consults for other pathogens/topics (*P* < .01). Consults pertaining to *M. genitalium* versus other topics were more likely to come from a private practice or women's health clinic versus other work settings (28% vs 8%, *P* < .01) (Figure 3).

## DISCUSSION

In response to a recent surge in STDCCN consults related to *M. genitalium*, we set out to quantify this phenomenon as well as characterize consult features that could suggest drivers of this surge and ultimately identify opportunities for provider outreach and education. We found a sharp rise in the volume of *M. genitalium* consults starting in 2023, which were disproportionately related to management in pregnant patients (often in the absence of reported symptoms) and more often submitted by clinicians located in a private practice or women's health center.

After a gradual rise beginning in 2021, *M. genitalium* consult volume increased dramatically in 2023, and currently comprises the second most common topic among all STDCCN consult submissions after syphilis. There are likely multiple factors driving this phenomenon. The high frequency of consults for treatment failure suggest that increasing *M. genitalium* resistance, which has been reported both in the USA and globally, is 1 potential driver [16, 17]. *M. genitalium* resistance to macrolides and the move away from azithromycin as a first line *M. genitalium* treatment regimen in the 2021 guidelines meant that treatment of *M. genitalium* in pregnant patients grew more challenging (with limited treatment options and sparse published treatment outcomes), which likely further drove consult volume. Also, growing provider awareness of this emerging STI following its inclusion in the CDC guidelines in 2015 and 2021, as well as availability of testing following the first FDA approval of a commercial *M. genitalium* assay in 2019, almost certainly contributed to increases in *M. genitalium* testing and detection of more infections that needed management by providers who may have had little experience in doing so. However, none of these explanations alone likely can fully explain the sudden change between 2022 and 2023. Of note, in May 2022, the FDA approved the first multiplex PCR panel [18] that bundled an *M. genitalium* NAAT with testing for *N. gonorrhoeae* and *C. trachomatis.* The subsequent surge in *M. genitalium* consults after this time suggests in part that use of this multiplex technology was also a driving force in new *M. genitalium* diagnoses, which reveals potential drawbacks inherent to use of these bundled tests for routine STI screening (since *M. genitalium* screening is not recommended by CDC).

The CDC recommends screening pregnant people for *N. gonorrhoeae* or *C. trachomatis* at the first prenatal visit, and again in the third trimester for some groups. Pregnant patients comprised more than half of clinical consults in our study and were more likely than nonpregnant female patients to not have specified symptoms. As the CDC does not recommend routine screening for *M. genitalium* in asymptomatic patients (including during pregnancy), this may reflect provider lack of familiarity with the guidelines and/or lack of awareness about appropriate use of multiplex PCR tests that include *M. genitalium*. Detection of *M. genitalium* during pregnancy presents a clinical challenge, as the first line recommended treatment regimen in the absence of having data confirming a macrolide sensitive strain is sequential doxycycline and moxifloxacin, both which have a contraindication for use in pregnancy due to concerns about potential fetal harm. The utility of azithromycin, the only *M. genitalium* treatment that is available in the United States and considered safe in pregnancy, is compromised by a high prevalence of macrolide resistance exceeding 40% [19], with known declining cure rates with the emergence of macrolide resistance [20]. The relationship of *M. genitalium* to adverse pregnancy and neonatal outcomes is incompletely understood, with reported potential association with preterm birth, but insufficient evidence to prove causality [6]. Given the known risks of the most effective antimicrobials and uncertain benefits of treatment, providers and pregnant patients are often left in an uncomfortable, uncertain situation when deciding how to manage a positive *M. genitalium* test result, underscoring the importance of provider education around avoiding routine *M. genitalium* screening in pregnancy.

Our study's findings are limited by the nature of the STDCCN. Individuals submitting consults decide how much (or how little) clinical information to include, and important details including symptoms, exam findings, other STI testing results, and type of *M. genitalium* diagnostic test used are not systematically collected. In particular, limited information about symptoms precludes definitive statements about appropriateness of testing. However, the overall constellation of findings strongly suggests specific factors underlying this increase in *M. genitalium* consult activity and highlight the need for additional research and clinical education on this highly prevalent pathogen.

We suggest that the NNPTC and other similar organizations increase efforts to educate providers about appropriate circumstances for *M. genitalium* testing, including appropriate use of multiplex STI testing panels. Outreach to individuals and organizations providing prenatal care should be a particular focus, to avoid the uncertainty and anxiety that may accompany an incidental diagnosis of *M. genitalium* infection in this setting. Most of all, more research is needed, both to clarify the effects of *M. genitalium* on pregnancy, neonatal, and fertility outcomes, and to develop more therapeutic agents for *M. genitalium* treatment, including those that do not have risk for fetal harm. With this information, future guidelines can optimally guide clinicians in the diagnosis and management of *M. genitalium*.
